# Barriers and facilitators to older adults’ acceptance of camera-based active and assisted living technologies: a scoping review protocol

**DOI:** 10.12688/openreseurope.16721.1

**Published:** 2023-11-24

**Authors:** Natalie An Qi Tham, Anne-Marie Brady, Martina Ziefle, John Dinsmore, visuAAL - Privacy-Aware and Acceptable Video-Based Technologies and Services for Active and Assisted Living

**Affiliations:** 1 Trinity Centre for Practice and Healthcare Innovation, School of Nursing and Midwifery, Trinity College Dublin, Dublin, Ireland; 2 Chair of Communication Science, Human-Computer Interaction Center, RWTH Aachen University, Aachen, Germany

**Keywords:** ambient assisted living, active and assisted living, computer vision, technology acceptance, older adults

## Abstract

**Objective:**

To identify and synthesize evidence on the barriers and facilitators to older adults’ acceptance of camera-based active and assisted living (AAL) technologies in the home.

**Introduction:**

Camera-based AAL technologies have been heralded as an important solution to population ageing. By leveraging state-of-the-art computer vision techniques, camera-based AAL technologies can secure greater levels of safety, health, and independence for older adults whilst benefiting their desires to age-in-place. However, these technologies face widespread rejection and are at present scarcely used. A critical first step toward enhancing older adults’ acceptance and uptake of camera-based AAL technologies is to understand the barriers and facilitators to their acceptance of said technology.

**Inclusion criteria:**

This review will consider primary studies reporting data on the barriers and facilitators to the acceptance of camera-based AAL technologies among community-dwelling older adults aged 60 and above. No date or language restrictions will be applied.

**Methods:**

Following JBI scoping review methodology, key electronic databases ( *e.g.*, MEDLINE, CINAHL, Embase, Web of Science, ACM Digital Library, IEEE Xplore) and the grey literature ( *e.g.*, Google Scholar) will be searched to locate both unpublished and published articles of relevance. Retrieved citations will undergo independent screening against pre-defined eligibility criteria. Data will be independently extracted and mapped to the Theoretical Domains Framework with guidance from a pre-piloted coding manual. Results will be presented in tabular form accompanied by a narrative summary of barriers and facilitators.

## Introduction

The speed and intensity at which populations are ageing has resulted in significant social, economic, and policy challenges. Countries worldwide have seen substantive increases in the proportional number of older adults (defined by the United Nations as individuals aged 60 years and above ^1^), and projections estimate that this demographic imbalance will only continue to intensify ^1^. Significant health system costs are accruing in what policymakers have dubbed a “demographic crisis” warranting immediate response ^2^. To this end, a plethora of government and industry-led initiatives have been enacted to facilitate an “ageing-in-place” agenda, which aims to prolong older adults’ ability to live safely and independently in their own homes and in so doing mitigate the accruing expenses of an increasingly aged population ^3^.

Substantial research interests have converged around the potential of active and assisted living (AAL) technologies to advance the ageing-in-place vision. AAL technologies are broadly defined as information and communication technologies designed to facilitate safe, healthy, and independent lives into old age in individuals’ preferred living environments ^4^. Distinguished by features of context-awareness, proactivity, and reactivity, AAL technologies are conceived to support older adults by myriad “intelligent” means. These include pre-empting dangerous situations such as falls and signaling for emergency assistance ^5^, and controlling features of the home environment to support older adults in their daily living ^6^. Some AAL technologies have even been conceived as a means for long-term health self-management by facilitating preventive actions to reduce the risk of chronic disease onset or exacerbation ^7^. Overall, AAL technologies are envisioned to secure safer, healthier, and more independent lives for older adults, enabling them to remain at home for longer than would otherwise be possible.

Within the corpus of AAL technologies, a new generation of camera-based technologies has emerged as an eminent recourse for technologically mediated care and support for older adults ^8^. Camera-based AAL technologies benefit from the maturity in computer vision research, where techniques have been developed to collect and interpret rich behavioral and contextual data supplied by cameras ^8^. A distinguishing feature of camera-based AAL technologies is their capacity to evaluate data at a level of precision and semantic detail that is difficult to achieve with vision-less sensors ^8^. For example, state-of-the-art cameras have been developed that can evaluate individuals for morphological indicators of chronic disease risk ( *e.g.*, obesity) and make subsequent recommendations to reduce this risk, such as suggestions to modify one’s diet or physical activity ^7^. Other camera-based AAL technologies have been developed that can infer emotional indicators of social isolation from individuals’ facial expressions, with a view to enact stimulatory means to improve mood and ameliorate episodic depression or anxiety ^9^. Given their perceptual breadth and depth, camera-based AAL technologies are well poised to advance the ageing-in-place paradigm.

Despite their forecasted appeal, the interventionist potential of camera-based AAL technologies has to date been underrealized. A research trend reveals that even where older adults are willing to adopt AAL technologies, this willingness precludes the use of cameras ^10^. While traditional models of technology acceptance have typically attributed this hesitancy to difficult-to-use or inaccessible technologies ^11^, contemporary scholarship alludes to a range of environmental, social, and psychological factors that impede the acceptance of AAL technologies. These include, for example, the desire to dissociate from the stigmatizing connotations of assisted living ^12^ and the perceived inadequacy of AAL technologies to subsume a necessarily human caregiver role ^13^. Most frequently discussed in the context of camera-based AAL technologies are the privacy implications of camera-based monitoring. This is because a monitored home is typically experienced as alienating, having been transformed from a place of comfort and refuge to one of disconcerting exposure and vulnerability ^14^. Indeed, in the unique context of camera-based AAL, feelings of “being watched” ^15^ or “spied on” ^15^ by unwanted entities often supplant older adults’ otherwise intact desires to receive technologically mediated care.

Importantly, however, these privacy concerns are not unyielding. There is evidence that older adults are willing to trade-off their privacy for AAL features that are deemed particularly beneficial, such as the ability to delay or altogether prevent the transition to long-term care ^16^. Other studies reveal that negative attitudes towards camera-based surveillance are attenuated among individuals with heightened needs for care ^17^. Presumably, for older adults for whom declining health is a genuine concern, privacy concessions are a necessary sacrifice for longer, healthier lives.

Overall, there exists a range of physical, social, psychological, and environmental factors that determine whether camera-based AAL technologies are appropriated or rejected by older adults. To date, however, these factors have received little attention. Preliminary searches of the Cochrane Database of Systematic Reviews, JBI Evidence Synthesis, and MEDLINE were conducted and no systematic or scoping reviews on the topic were identified. Where both the barriers and facilitators to older adults’ acceptance of AAL technologies have been systematically examined, camera-based AAL technologies do not feature meaningfully in the types of technologies under study ^18^. Understanding the factors that drive or hinder older adults’ acceptance of camera-based AAL technologies is paramount towards informing the development of more acceptable, and thus more widely used, camera-based AAL technologies.

### Conceptual framework

Any attempt to increase individuals’ technology acceptance is an endeavor in behavior change, as the aim is to change individuals’ orientations towards technology from ones characterized by hesitancy and resistance to ones of endorsement and willing uptake.

Psychological theories offer important utility for changing behavior. Theories of behavior, in particular, aid in explaining and predicting behavior by specifying why, when, and how a particular behavior ( *e.g.*, acceptance of AAL technologies) does or does not occur, as well as key sources of influence to be targeted in order to change the behaviour ^19^. In so doing, behavioral theories inform the systematic development behavior change interventions, such as interventions designed to enhance older adults’ acceptance of camera-based AAL technologies ^20^.

The Theoretical Domains Framework (TDF) ^21^ is a framework against which the determinants of older adults’ acceptance of camera-based AAL technologies might be suitably conceptualized to benefit behavior change efforts. The TDF specifies 14 theoretical constructs important for understanding and changing behavior: *knowledge*; *skills*; *social or professional role and identity; beliefs about capabilities; optimism; beliefs about consequences; reinforcement; intentions; goals; memory, attention, and decision processes; environmental context and resources; social influences; emotions;* and *behavioral regulation*. Given its theoretical breadth, a TDF-based synthesis of behavioral determinants will aid in uncovering the range of factors that promote or impede older adults’ acceptance of camera-based AAL technologies.

## Review question

What are the barriers and facilitators to older adults’ acceptance of camera-based AAL technologies?

## Methods

The scoping review will be conducted in accordance with the JBI methodology for scoping reviews ^22^ and reported in accordance with the Preferred Reporting Items for Systematic Reviews and Meta-Analyses extension for Scoping Reviews (PRISMA-ScR) ^23^. A scoping review was deemed appropriate as it provides a rigorous framework for synthesizing emerging evidence within a fragmented research space ^24^. Given the incipient and methodologically heterogenous nature of scholarship pertaining to older adults’ acceptance of camera-based AAL technologies, a scoping review is therefore likely to return the greatest informational yield by allowing a rich examination of the nature and extent of, and preconditions for, older adults’ acceptance of camera-based AAL technologies.

### Participants

This review will consider studies with a population of older adults aged ≥ 60 years, or that include a subgroup analysis of older adults aged ≥ 60 years. Studies that include other stakeholder groups involved in the care of older adults ( *e.g.*, healthcare professionals, caregivers) will be excluded unless data specific to older adults can be extracted separately.

### Concept

Studies will be considered if they involve discussion of camera-based AAL technologies, operationalized as any technology that incorporates the use of at least one camera in supporting safer, healthier, and more independent lives into old age ^8, 25^. A defining feature of camera-based AAL technologies is a central point of processing to which camera streams are transmitted, stored and analysed ^25^. Therefore, studies will be excluded if the examined technology is devoid of such central processing points, such as videophony telehealth systems where cameras simply facilitate real-time communication between patient and provider. For conceptual parsimony, studies must investigate cameras that are embedded “in the environment” such as wall-hung cameras; Studies examining wearable cameras will be excluded. Studies must additionally include data on barriers and facilitators to acceptance of the technology under study. Studies that evaluate proxy measures of acceptance, such as intention to use or actual use of the technology, will be considered.

### Context

Studies investigating camera-based AAL technologies in private residential settings such as participants’ own homes or residences in retirement communities will be included. This criterion reflects the central thrust of the ageing-in-place paradigm, which aims to decentralize the provision of care for older adults from non-private acute, sub-acute, or institutional settings to the home environment ^26^. Therefore, studies that sample from non-private settings such as assisted living facilities or nursing homes will be excluded.

### Types of sources

Studies of quantitative, qualitative, or mixed methods designs that present original data will be considered for inclusion. Non-empirical or secondary articles such as reviews, study protocols, editorials, commentaries, opinion pieces, or other publication types that do not present original data will be excluded but will nevertheless undergo reference list screening for potentially eligible primary-level articles.

### Search strategy

The search strategy will aim to locate both published and unpublished articles. To ensure wide coverage of the literature, six key databases will be searched: MEDLINE, CINAHL, Embase, Web of Science, IEEE Explore Digital Library, and ACM Digital library. Initial limited searches of MEDLINE were undertaken to identify articles on the topic. The text words contained in the titles and abstracts of relevant articles and the MeSH terms describing the articles were used to develop a full MEDLINE search strategy. Given the limited scope of research focusing specifically on camera-based AAL technologies as a major topic, methodologic filters that incorporate terms relating to cameras ( *e.g.*, “cameras”, “video”, “visual”, “vision”) may prematurely exclude relevant articles. Therefore, the search strategy excludes reference to cameras and other associated terms for enhanced sensitivity. A broad four-concept search strategy has been developed with guidance from an expert subject librarian and features a combination of index terms and keyword search strings centered around: (i) active and assisted living, (ii) older adults, (iii) the home setting, and (iv) acceptance. Following refinement, the MEDLINE search strategy (see Table 1) will be adapted for each database as appropriate.

To achieve the purposive breadth entailed in scoping reviews, searches will be conducted across the Google Scholar grey literature database to ensure that no relevant publications are missed. The reference lists of all retrieved records will be hand-screened to identify other potentially eligible documents. Where necessary, authors of papers will be contacted to obtain potentially relevant unpublished studies. Following piloting and refinement of search strategies, the lead author (NAQT) will run searches across all databases. No date or language restrictions will be imposed.

**Table 1.** MEDLINE Search Strategy. Search conducted on March 31, 2023

S1	AB (aged OR ageing OR aging OR elder* OR frail* OR geriatr* OR gerontol* OR “later in life*” OR “later life*” OR “old age” OR “old* adult*” OR “old* individual*” OR “old* people*” OR “old* person*” OR “old* vulnerable” OR retire* OR senescent OR senile OR senior* ) OR TI (aged OR ageing OR aging OR elder* OR frail* OR geriatr* OR gerontol* OR “later in life*” OR “later life*” OR “old age” OR “old* adult*” OR “old* individual*” OR “old* people*” OR “old* person*” OR “old* vulnerable” OR retire* OR senescent OR senile OR senior* )	1,367,760
S2	AB (accept* OR abandon*OR adhere* OR adopt* OR assimilat* OR attitud* OR belief* OR compliance OR comply OR consideration* OR expect* OR experience* OR fear* OR feel* OR integrat* OR intent* OR non-use OR nonuse OR opinion* OR perceive* OR perception* OR perspective* OR preference* OR reason* OR reject* OR satisf* OR view*) OR TI (abandon* OR accept* OR adhere* OR adopt* OR assimilat* OR attitud* OR belief* OR compliance OR comply OR consideration* OR expect* OR experience* OR fear* OR feel* OR integrat* OR intent* OR non-use OR nonuse OR opinion* OR perceive* OR perception* OR perspective* OR preference* OR reason* OR reject* OR satisf* OR view*)	5,760,259
S3	AB (“age in place” OR “age-in-place” OR “ageing at home” OR “ageing from home” OR “ageing in place” OR “ageing-in- place” OR “aging at home” OR “aging from home” OR “aging in place” OR “aging-in-place” OR “at home” OR at-home OR “community dwelling” OR “community-dwelling*” OR domicile OR dwelling* OR house* OR home* OR home-based OR home-environment* OR “home environment*” OR “in home” OR in-home OR “private home*” OR residenc* OR residential OR “retirement communit*” OR “retirement home*” OR “retirement village*”) OR TI (“age in place” OR “age- in-place” OR “ageing at home” OR “ageing from home” OR “ageing in place” OR “ageing-in-place” OR “aging at home” OR “aging from home” OR “aging in place” OR “aging-in-place” OR “at home” OR at-home OR “community dwelling” OR “community-dwelling*” OR domicile OR dwelling* OR house* OR home* OR home-based OR home-environment* OR “home environment*” OR “in home” OR in-home OR “private home*” OR residenc* OR residential OR “retirement communit*” OR “retirement home*” OR “retirement village*”)	947,526
S4	S1 AND S2 AND S3	54,229
S5	((MH “Ambient Intelligence” OR AAL OR “activity recognition” OR “activity detection” OR “ambient assist*” OR ambient- assist* OR “ambient intelligence” OR “ambient intelligent” OR AmI OR “assist* living” OR eldercare OR “independent living” OR monitor* OR “motion detect*” OR “motion recognit*” OR “smart building*” OR “smart home*” OR “smart house*” OR “smart residence*” OR surveil* OR “ubiquitous comput*” OR “ubiquitous monitor*”) N2 (technolog* OR system* OR device* OR application*)	594,236
S6	S4 AND S5	3,408

### Study/Source of evidence selection

All identified records will be collated and imported into EndNote 20.1 (Clarivate Analytics, PA, USA). Following de-duplication, articles will undergo a two-phase screening process using Covidence (Veritas Health Innovation, Melbourne, Australia). PICO Portal is an alternative review manager with a free version that can perform an equivalent function. The titles and abstracts will be screened by two independent reviewers for assessment against the inclusion criteria. Obviously irrelevant studies will be excluded from further review. The remaining studies will then undergo full-text screening by two independent reviewers (NAQT and a second reviewer) and only those meeting the eligibility criteria will be retained. During this phase, particular focus will be exercised to ascertain the presence of cameras in the technology under study. Any discrepant articles will undergo inter-reviewer discussion and where consensus cannot be achieved, arbitration will be sought from a third reviewer (JD) who has expertise in theory-informed health behavior change. To ensure process transparency and reproducibility, reasons for the exclusion of full-text articles will be recorded and reported. Search results will be reported in full in the final review and visualized using a PRISMA flow diagram ^23^.

### Data extraction

Data extraction will be guided by a modified JBI extraction tool (see Table 2). In addition to study classifiers ( *e.g.*, authors, publication year, country) and characteristics ( *e.g.*, aim, design, participants), articles will be extracted for information relating to the camera-based AAL technology under study ( *e.g.*, type of camera used, context of usage), outcome measures ( *e.g.*, acceptance, actual technology usage), and barriers and facilitators. Barriers and facilitators will be operationalized as any factor, characteristic, view, or belief that impedes or enables older adults’ acceptance of camera-based AAL technologies ^27^. The extraction form will be adjusted as necessary during the extraction process to ensure comprehensive representation of all data sources and relevance of the extracted data to the research question.

Data on barriers and facilitators will be extracted from the results and discussion sections of included studies. Extracted data will likely vary based on study design and the type of data presented and will include (i) results of statistical analyses; (ii) verbatim quotations from participants and/or authors; and (iii) narrative descriptive summaries of results per the authors’ original report. Study authors will be contacted in cases of incomplete data or uncertainty.

Data extraction will be independently piloted on a subset of studies with discrepancies resolved through inter-reviewer discussion and consultation with the third arbiter JD where appropriate. Thereafter, NAQT will undertake data extraction across all remaining studies.

**Table 2.** Data extraction instrument.

**Study reference**	
Author(s)	Authors of the paper as surname and initials
Publication date	Date that paper was published
Title	Title of the study
**Study context**	
Research question/aim	Summary of the stated aim of the study
Location of origin	Country in which the affiliation of the first author is based
Target population	List the specific population studied ( *e.g.*, older adults, older adults and their family members)
**Study methodology**	
Study design	List the design of the study ( *e.g.*, qualitative, quantitative, mixed-methods)
Data collection method	List the method by which data was collected ( *e.g.*, interviews, focus groups, questionnaires)
**Study participants**	
Sample size	Give exact *N* of participants who completed the study
Sample size of older adult population	Give exact *N* of older adult participants who completed the study
Participant characteristics	List demographics of older adult participants including age, gender, etc.
**AAL technology under investigation**	
Details of technology under evaluation	List the types of technology under study ( *e.g.*, cameras, motion sensors)
Details of camera-based AAL technology under evaluation	List the type(s) of camera used in the camera-based AAL technology under study, where specified ( *e.g.*, standard Red-Green-Blue camera, depth camera, thermal camera, etc.)
Context of evaluation and/ or use	List the setting in which the camera-based AAL technology has been, or is expected to be, implemented
**Data analysis**	
Method of data analysis	List the methods by which data was analyzed ( *e.g.*, frequency analysis, thematic analysis)
**Study findings**	
Summary of main findings according to author(s)	Extract main findings as reported by study author(s)
Outcome(s)	Extract outcome(s) of study as related to acceptance of the camera-based AAL technology under study. Include details such as how acceptance is defined and measured ( *e.g.*, acceptance, willingness-to-use, actual usage)
Key findings that relate to the scoping review question	Extract the name of the construct identified as a determinant of acceptance (or non-acceptance) of the camera-based AAL technology under study. Accordingly, list whether the construct is a barrier or facilitator to acceptance. Where applicable, extract information relating to the prevalence ( *i.e.*, percentage of participants identifying factor as barrier/facilitator to acceptance) and strength of the relationship ( *i.e.*, measure of association between identified barrier/facilitator and acceptance)
TDF Domain	Code each determinant ( *i.e.*, barrier and/or facilitator) to TDF domain(s) deemed relevant

### Data transformation

A convergent integrated approach ^28^ will be used to integrate and synthesize qualitative and quantitative evidence. Quantitative data will be converted into textual descriptions using declarative descriptions and narrative interpretations in ways that answer the review question, as specified in JBI methodological guidance for mixed-methods reviews ^28^. For instance, where quantitative studies report lower acceptance rates among older adults with lower ( *versus* higher) income, this will be recorded as the statement: “Cost is a barrier to acceptance.”

### Data mapping

Extracted barriers and facilitators will be deductively mapped onto the TDF domains using directed content analysis ^29^. Coding consistency and accuracy will be facilitated by a coding manual developed in consultation with an expert in health behaviour change (JD) to generate consensus in interpretation and categorization of barriers and facilitators. The coding manual will derive from the original TDF ^21^ and will include theoretical definitions of each of the 14 TDF domains as well as decision rules specifying example items to code under each domain (see Table 3).

**Table 3.** Data coding manual.

TDF domain and content	Constructs	Decision Rules
**Knowledge:** An awareness of the existence of something	Knowledge (including knowledge of condition): An awareness of the existence of something	Consider coding to this domain: • Discussion relating to older adults’ knowledge and understanding (or lack thereof) of how to use camera-based AAL technologies. Inappropriate coding to this domain: • Discussion relating to personalized accounts of hypothetical behavior - *e.g.*, statements such as “I would educate myself on how to use the technology” should be coded at “Behavioral Regulation” instead.
	Procedural knowledge: Knowing how to do something	
	Knowledge of task environment: Knowledge of the social and material context in which a task is undertaken	
**Skills:** an ability or proficiency acquired through practice	Skills: An ability or proficiency acquired through training and/or practice	Consider coding to this domain: • Discussion about the skills (or lack thereof) employed by older adults to use camera-based AAL technologies. Inappropriate coding to this domain: • Discussion relating to older adults’ confidence in their ability to use camera-based AAL technologies should be coded at “Beliefs about capabilities” instead.
	Skills development: The gradual acquisition or advancement through progressive stages of an ability or proficiency acquired through training and practice	
	Competence: One’s repertoire of skills, and ability especially as it is applied to a task or set of tasks	
	Ability: Competence or capacity to perform a physical or mental act. Ability may be either unlearned or acquired by education and practice	
	Interpersonal skills: An aptitude enabling a person to carry on effective relationships with others, such as an ability to cooperate, to assume appropriate social responsibilities or to exhibit adequate flexibility	
	Practice: Repetition of an act, behavior, or series of activities, often to improve performance or acquire a skill	
	Skills assessment: A judgement of the quality, worth, importance. Level or value of an ability or proficiency acquired through training and practice	
**Social/Professional role and** ** identity:** A coherent set of behaviors and displayed personal qualities of an individual in a social or work setting	Professional identity: The characteristics by which an individual is recognized relating to, connected with, or befitting a particular profession	Consider coding to this domain: • Discussion relating to older adults’ perceived need (or lack thereof) for camera-based AAL technologies. • Discussion relating to older adults’ belief about the stigmatizing qualities of camera-based AAL technologies. • Discussion relating to how older adults’ self-identity - *e.g.*, as “old-fashioned” - impacts their acceptance of camera-based AAL technologies. • Discussion relating to older adults’ belief that using camera-based AAL technologies threatens their autonomy and/or dignity. Inappropriate coding to this domain: • Statements relating to social relationships that influence older adults’ acceptance of camera-based AAL technologies – *e.g.*, descriptions of older adults’ need for social approval from peers or family members and how this influences their acceptance decisions - should be coded at “Social Influences” instead.
	Professional role: The behavior considered appropriate for a particular kind of work or social position	
	Social identity: The set of behavioral or personal characteristics by which an individual is recognizable [and portrays] as a member of a social group	
	Identity: An individual’s sense of self defined by a) a set of physical and psychological characteristics that is not wholly shared with any other person and b) a range of social and interpersonal affiliations ( *e.g.*, ethnicity) and social roles.	
	Professional boundaries: The bounds or limits relating to, or connected with a particular profession or calling	
	Professional confidence: an individual’s belief in his or her repertoire of skills and ability especially as it is applied to a task or set of tasks.	
	Group identity: the set of behavioral or personal characteristics by which an individual is recognizable [and portrays] as a member of a group	
	Leadership: The processes involved in leading others, including organizing, directing, coordinating, and motivating their efforts toward achievement of certain group or organization goals	
	Organizational commitment: An employee’s dedication to an organization and wish to remain part of it. Organizational commitment is often described as having both an emotional or moral element and a more prudent element	
**Beliefs about capabilities:** Acceptance of the truth, reality, or validity about an ability, talent, or facility that a person can put to constructive use	Self-confidence: Self-assurance or trust in one’s own abilities, capabilities, and judgement	Consider coding to this domain: • Descriptions of older adults’ sense of aptitude for, or confidence in, using camera-based AAL technologies ( *i.e.*, self-efficacy). • Descriptions of older adults’ perceived control over the operations of camera-based AAL technologies ( *e.g.*, turning cameras on/off, deciding where, when, and how recording takes place, and to whom information should be transmitted). • Descriptions of older adults’ perceived control over the decision of whether to use camera-based AAL technologies. Inappropriate coding to this domain • Descriptions of older adults’ beliefs about the capabilities of other individuals ( *e.g.*, family members, informal/formal caregivers) to use camera-based AAL technologies.
	Perceived competence: An individual’s belief in her or her ability to learn and execute skills	
	Self-efficacy: An individual’s capacity to act effectively to bring about desired results, as perceived by the individual	
	Perceived behavioral control: an individual’s perception of the ease or difficulty of performing the behavior of interest	
	Beliefs: The thing believed; the proposition or set of propositions held true	
	Self-esteem: The degree to which the qualities and characteristics contained in one’s self-concept are perceived to be positive	
	Empowerment: The promotion of the skills, knowledge, and confidence necessary to take great control of one’s life as in certain educational or social schemes; the delegation of increase decision- making powers to individuals or groups in a society or organization	
	Professional confidence: An individual’s beliefs in his or her repertoire of skills, and ability, especially as it is applied to a task or set of tasks.	
**Optimism:** The confidence that things will happen for the best or that desired goals will be attained	Optimism: The attitude that outcomes will be positive and that people’s wishes or aims will be ultimately fulfilled	Consider coding to this domain: • Descriptions of older adults' optimism regarding their current health optimism regarding their current health and/or the reality of their ageing – *e.g.*, descriptions of older adults’ belief that they are “younger” or “healthier” than they really are – and how this impacts their acceptance of camera-based AAL technologies. Inappropriate coding to this domain: • Description of other people’s ( *e.g.*, family members, healthcare professionals) levels of optimism about the effectiveness and/or usefulness of camera-based AAL technologies.
	Pessimism: The attitude that things will go wrong and that people’s wishes or aims are unlikely to be fulfilled	
	Unrealistic optimism: the inert tendency for humans to over-rate their own abilities and chances of positive outcomes compared to those of other people	
**Beliefs about Consequences:** Acceptance of the truth, reality, or validity about outcomes of a behavior in a given situation	Beliefs: The thing believed; the proposition or set of propositions held true	Consider coding to this domain: • Description of older adults’ beliefs about the potential negative outcomes that may result from their use of camera-based AAL technologies such as privacy infringements, technical issues (e.g., false alarms), etc. • Description of older adults’ beliefs about the positive outcomes that may result from their use of camera-based AAL technologies – increased health, wellbeing, safety, independence, longevity, etc. • Description of older adults’ belief that camera-based AAL technologies confer (or do not confer) utility. • Description of older adults’ belief about outcomes relating to the data that will be collected, processed, and transmitted by camera- based AAL technologies – *e.g.*, beliefs about whether images and/or recordings undergo (or do not undergo) processing for enhanced privacy. • Note: outcomes can be theoretical or the result of actual experience. Inappropriate coding to this domain: • Description relating to an anticipated outcome of using camera- based AAL technologies that is based on the beliefs that older adults have about themselves – *e.g.*, description of older adults’ belief that camera-based AAL technologies will be of little to no utility to them because they see themselves as “young and healthy” should be coded to “Social/Professional Role and Identity” instead.
	Outcome expectancies: Cognitive, emotional, behavioral, and affective outcomes that are assumed to be associated with future or intended behavior. These assumed outcomes can either promote or inhibit future behaviors.	
	Characteristics of outcome expectancies: Characteristics of the cognitive, emotional, and behavioral outcomes that individuals believe are associated with future or intended behaviors and that are believed to either promote or inhibit these behaviors. These include whether they are sanctions/rewards, proximal/distal, valued/not valued, probable/improbable. Salient/not salient, perceived risks or threats.	
	Anticipated regret: A sense of the potential negative consequences of a decision that influences the choice made: for example, an individual may decide not to make an investment because of the feelings associated with an imagined loss	
	Consequents: An outcome behavior in a given situation	
**Reinforcement:** Increasing the probability of a response by arranging a dependent relationship, or contingency, between the response and a given stimulus	Rewards (proximal/distal, valued/ not valued, probable/improbable): Return or recompense made to, or received by a person contingent on some performance.	Consider coding to this domain: • Description of how older adults’ previous usage of (assistive) technology impacts their current acceptance of camera-based AAL technologies. • Description of how older adults’ previous experiences ( *e.g.*, prior adverse health events, prior receipt of care) impact their current acceptance of camera-based AAL technologies. Inappropriate coding to this domain: • Description of outcomes that older adults expect to result from using camera-based AAL technologies that are not contingent rewards – *e.g.*, description of older adults’ belief that using camera-based AAL technologies will relieve their family members’ caregiving burdens should be coded to “Social Influences” instead.
	Incentives: An external stimulus, such as condition or object, that enhances or serves as a motive for behavior	
	Punishment: The process in which the relationship between the response and stimulus or circumstance results in the response becoming less probable; a painful, unwanted, or undesired event or circumstance imposed as a penalty on a wrongdoer	
	Consequents: An outcome of behavior in a given situation	
	Reinforcement: A process in which the frequency of a response is increased by a dependent relationship or contingency with a stimulus	
	Contingencies: A conditional probabilistic relation between two events. Contingencies may be arranged via dependencies or they may emerge by accident	
	Sanctions: A punishment or other coercive measure, usually administered by a recognized authority, that is used to penalize and deter inappropriate or unauthorized actions.	
**Intentions:** A conscious decision to perform a behavior or a resolve to act in a certain way	Stability of intentions: ability of one’s resolve to remain in spite of disturbing influences	Consider coding to this domain: • Description of older adults’ personal intent, motivation, or inclination to use camera-based AAL technologies. • Note: Use of the 1st person “I will”, “I would” are strong indications to consider coding at this domain. • Note: Indicators of intention should be explicit and not inferred. Statements should therefore directly reflect older adults’ intention and/or motivation, rather than the reasons underpinning this intention. Inappropriate coding to this domain: • Description of how older adults choose between two or more alternatives in order to reach an intended outcome – *e.g.*, description of how older adults prioritize their ability to remain at home over and above preserving their privacy should be coded at “Memory, Attention, and Decision Processes” instead.
	Stages of Change model: A model that proposes that behavior change is accomplished through five specific stages	
	Transtheoretical model and stages of change: a five-stage theory to explain changes in people’s health behavior. It suggests that change takes time, that different interventions are effective at different stages, and that there are multiple outcomes occurring across the stages	
**Goals:** Mental representations of outcomes or end states that an individual wants to achieve	Goals (distal/proximal): Desired state of affairs of a person or system, these may be closer (proximal) or further away (distal)	Consider coding to this domain: • Description of older adults’ acceptance of camera-based AAL technologies in relation to a distinct and identifiable endpoint - *e.g.*, description of older adults’ decision to use camera-based AAL technologies in order to age-in-place and avoid institutionalization should be coded here. This differs from “Intentions” where older adults may describe their resolve to use the technology without any specific reference to an endpoint. Inappropriate coding to this domain: • Description of how older adults prioritize one anticipated outcome of using camera-based AAL technologies over another may be more appropriately coded elsewhere, especially if no specific reference is made to target endpoints – *e.g.*, statements relating to trade-offs made by older adults between preserving their privacy and having enhanced health and independence should be coded at “Memory, Attention, and Decision Processes” instead.
	Goal priority: Order of importance or urgency of end state toward which one is striving	
	Goal/target setting: A process that establishes specific time-based behavioral targets that are measurable, achievable, and realistic	
	Goals (autonomous/controlled): The end state toward which one is striving: the purpose of an activity or endeavor. It can be identified by observing that a person ceases or changes their behavior upon attaining this state; proficiency in a task to be achieved within a set period of time	
	Action planning: The action or process of forming a plan regarding a thing to be done or a deed	
	Implementation intention: The plan that one creates in advance of when, where, and how one will enact a behavior	
**Memory, Attention and Decision Processes:** The ability to retain information, focus selectively on aspects of the environment and choose between two or more alternatives	Memory: The ability to retain information or a representation of a past experience, based on the mental processes of learning or encoding retention across some interval of time, and retrieval or reactivation of the memory; specific information of a specific task	Consider coding to this domain: • Description of the cognitive cost-benefit analyses that older adults engage in when contemplating usage of camera-based AAL technologies. • Description of cognitive processes involved when older adults choose between two or more alternative outcomes in relation to using camera-based AAL technologies – *e.g.*, trade-offs between preserving their privacy and ageing-in-place. • Description of older adults’ decisions regarding the timing of their usage of camera-based AAL technologies – *e.g.*, description of a willingness to use the technology in the future but not now. Inappropriate coding to this domain • Description of older adults’ beliefs about their own decisional control over whether to use camera-based AAL technologies should be coded at “Beliefs about Capabilities” instead.
	Attention: A state of awareness in which the senses are focused selectively on aspects of the environment and the central nervous system is in a state of readiness to respond to stimuli	
	Attention control: The extent to which a person can concentrate on relevant cues and ignore all irrelevant cues in a given situation	
	Decision making: The cognitive process of choosing between two or more alternatives, ranging from the relatively clear-cut to the complex	
	Cognitive overload/tiredness: The situation in which the demands placed on a person by mental work are greater than a person’s mental abilities	
**Environmental Context and Resources:** Any circumstance of a person's situation or environment that discourages or encourages the development of skills and abilities, independence, social competence, and adaptive behavior	Environmental stressors: External factors in the environment that cause stress	Consider coding to this domain: • Description of the obtrusiveness (or lack thereof) of camera-based AAL technologies, as perceived by older adults. • Description of the affordability (or lack thereof) of camera-based AAL technologies, as perceived by older adults. • Description of the ease-of-use (or lack thereof) of camera-based AAL technologies, as perceived by older adults • Description of the availability (or lack thereof) of resources ( *e.g.*, Internet connection) to facilitate usage of camera-based AAL technologies. • Description of how camera-based AAL technologies are seen as integrating (or failing to integrate) with older adults’ existing lifestyles. Inappropriate coding to this domain: • Description of the actions taken by older adults in order to secure the necessary resources required to use camera-based AAL technologies should be coded at “Behavioral Regulation” instead.
	Resources/material resources: Commodities and human resources used in enacting a behavior	
	Organizational culture/climate: A distinctive pattern of thought and behavior shared by members of the same organization and reflected in their language, values, attitudes, beliefs, and customs	
	Salient events/critical incidents: Occurrences that one judges to be distinctive, prominent, or otherwise significant	
	Personal environment interaction: Interplay between the individual and their surroundings	
	Barriers and facilitators: In psychological contexts, barriers/facilitators are mental, emotional, or behavioral limitations/strengths in individuals or groups	
**Social influences:** Those interpersonal processes that can cause individuals to change their thoughts, feelings, or behaviors	Social pressure: the exertion of influence on a person or group by another person or group	Consider coding to this domain: • Description of older adults’ preference for human-provided care and interaction compared to technologically mediated care and interaction. • Description of older adults’ concern about how camera-based AAL technologies may create burdens for their caregivers. • Description of older adults’ belief that using camera-based AAL technologies will relieve the burdens faced by their family members, caregivers and/or healthcare professionals. • Description of older adults’ belief that using camera-based AAL technologies will allow them to build stronger social relationships and/or expand their social networks. • Description of how camera-based AAL technologies ( *e.g.*, social robots) are seen as social companions. • Description of how older adults’ acceptance of camera-based AAL technologies is influenced by the opinions or behaviors of others - *e.g.*, family members, community peers, healthcare professionals. • Description of how the presence (or lack thereof) of social support ( *e.g.*, spousal support) influences older adults’ acceptance of camera-based AAL technologies. • Discussion relating to how instructions and/or guidance from others impact older adults’ acceptance of camera-based AAL technologies – *e.g.*, descriptions of how older adults accept the technology due to medical directives by healthcare professionals. Inappropriate coding to this domain: • Description of older adults’ belief that camera-acquired data will be transmitted only to certain authorized individuals ( *e.g.*, family members, healthcare professionals) should be coded at “Beliefs about Consequences” instead, as this relates more to the perceived consequences of using the technology.
	Social norms: Socially determined consensual standards that indicate a) what behaviors are considered typical in a given context and b) what behaviors are considered proper in the context	
	Group conformity: The act of consciously maintaining a certain degree of similarity to those in your general social circles	
	Social comparisons: The process by which people evaluate their attitudes, abilities, or performance relative to others	
	Group norms: Any behavior, belief, attitude, or emotional reaction held to be correct or acceptable by a given group in society	
	Social support: The apperception or provision of assistance or comfort to others, typically in order to help them cope with a variety of biological, psychological, and social stressors. Support may arise from any interpersonal relationship in an individual’s social network, involving friends, neighbors, religious institutions, colleagues, caregivers of support groups	
	Power: The capacity to influence others, even when they try to resist this influence	
	Intergroup conflict: Disagreement or confrontation between two or more groups and their members. This may involve physical violence, interpersonal discord, or psychological tension	
	Alienation: Estrangement from one's social group; a deep-seated sense of dissatisfaction with one's personal experiences that can be a source of lack of trust in one's social or physical environment or in oneself; the experience of separation between thoughts and feelings	
	Group identity: The set of behavioral or personal characteristics by which an individual is recognizable [and portrays] as a member of a group	
	Modelling: In developmental psychology the process in which one or more individuals or other entities serve as examples (models) that a child will copy	
**Emotion:** A complex reaction pattern, involving experiential, behavioral, and physiological elements, by which the individual attempts to deal with a personally significant matter or event	Fear: An intense emotion aroused by the detection of imminent threat, involving an immediate alarm reaction that mobilizes the organism by triggering a set of physiological changes	Consider coding to this domain: • Description of the emotions experienced or anticipated by older adults in relation to being monitored by cameras – *e.g.*, anxiety, fear, anger, etc. • Description of the emotions experienced or anticipated by older adults in relation to being seen by others as users of camera-based AAL technologies – *e.g.*, embarrassment, shame, humiliation, etc. • Description of how fearful, anxious, or negative attitudes towards technology influences older adults’ acceptance of camera-based AAL technologies. • Description of how experiencing particular emotions ( *e.g.*, fear of falling, fear of being without help) influences older adults’ acceptance of camera-based AAL technologies. Inappropriate coding to this domain: • Description of how other people’s emotions influence older adults’ acceptance of camera-based AAL technologies – *e.g.*, descriptions of how older adults use camera-based AAL technologies because they do not want their family members to feel worried should be coded at “Social Influences” instead.
	Anxiety: A mood state characterized by apprehension and somatic symptoms of tension in which an individual anticipates impending danger, catastrophe, or misfortune.	
	Affect: An experience or feeling of emotion, ranging from suffering to elation, from the simplest to the most complex sensations of feelings, and from the most normal to the most pathological emotional reactions.	
	Stress: A state of physiological or psychological response to internal or external stressors	
	Depression: A mental state that presents with depressed mood, loss of interest or pleasure, feelings of guilt or low self-worth, disturbed sleep or appetite, low energy, and poor concentration	
	Positive/negative affect: The internal feeling/state that occurs when a goal has/has not been attained. A source of threat has/has not been avoided, or the individual is/is not satisfied with the present state of affairs	
	Burn-out: Physical, emotional, or mental exhaustion, especially in one’s job or career, accompanied by decreased motivation, lowered performance and negative attitudes towards oneself and others	
**Behavioral Regulation:** Anything aimed at managing or changing objectively observed or measured actions	Self-monitoring: A method used in behavioral management in which individuals keep a record of their behavior, especially in connection with efforts to changes or regulate the self; a personality trait reflecting an ability to modify one’s behavior in response to a situation	Consider coding to this domain: • Description of the self-regulatory strategies employed by older adults that are aimed at facilitating their own use of or sustained engagement with camera-based AAL technologies. • Description of the actions taken by older adults in order to secure the necessary resources to facilitate their own use of or sustained engagement with camera-based AAL technologies. Inappropriate coding to this domain: • Description of older adults’ belief that camera-based AAL technologies can help them to regulate their own behavior – *e.g.*, descriptions of older adults’ belief that using camera-based AAL technologies can facilitate their self-management of chronic disease should be coded at “Belief about Consequences” instead, as this relates more to the perceived consequences of using the technology.

Coding will be independently piloted on a subset of studies, using inter-reviewer discussion and third-party arbitration to resolve discrepant codes. Thereafter, NAQT will undertake coding across all remaining studies. Barriers and facilitators may be coded to more than one domain if deemed appropriate. Any barrier or facilitator that is deemed unsuitable for TDF mapping will be collated into an “Others” domain. Thereafter, inductive analysis ^30^ will be used to further specify pertinent themes in each domain by NAQT and will undergo review by JD to ensure that themes are appropriately coded and sufficiently distinct.

### Data analysis and presentation

Descriptive study data will be presented within evidence tables. Key findings relating to barriers and facilitators will be narratively synthesized and thematically categorized by TDF domain in a manner that aligns with the review question. Barriers and facilitators will also be summarized in tabular format with illustrative quotations for greater elaboration. Any gaps in the literature will be examined and areas for future research will be outlined. Results of the review will be disseminated through peer-reviewed journals and conference presentations.

## Study status

As of October 2023, the scoping review is in the data mapping and data analysis stage. We expect that the review will be completed by January 2024.

## Ethics and consent

Ethical approval and consent were not required.

## Data Availability

No data are associated with this article.
