# Supplementary material for: Barriers and facilitators to older adults’ acceptance of camera-based active and assisted living technologies: a scoping review protocol
Source: Open Res Eur. 2023 Nov 24;3:210. [Version 1] doi: 10.12688/openreseurope.16721.1 (PMC10882197; doi:10.12688/openreseurope.16721.1)
Supplement: Peer review history

# Reviewer report: Reviewer response for version 1

**DOI:** 10.21956/openreseurope.18060.r38267

**Published:** 2024-03-04

**Authors:** Bruna Maria Vittoria Guerra

**Affiliations:** 1 University of Pavia, Pavia, Italy

The review is aimed at analyzing primary studies that delve into the factors influencing the acceptance of camera-based AAL technologies among older adults aged 60 and above who reside in community settings. It explores data on barriers and facilitators to the adoption of these technologies, with no constraints on language or publication date. While the review is well-written, there are a few comments that the authors may wish to consider.

1. The authors have opted to employ the Theoretical Domains Framework (TDF) as their chosen theoretical framework for the review. However, they have not adequately justified why they have selected this particular framework over others.
2. The authors touched upon the privacy concerns associated with camera-based AAL technologies. However, they did not explore the potential of RGB-D cameras, which offer depth imaging and skeletal data outputs, ensuring a completely privacy-preserving approach. I believe that the inclusion of this argument could significantly enhance the comprehensiveness of their analysis.
3. Did the authors solely explore databases containing data on camera-based technologies, or did they also include hybrid solutions incorporating ambient sensors and/or wearables?
4. Are there any comparable studies in the existing literature? If so, could the authors provide a comparison with the reviewed work?
5. Could the authors include additional review questions for further exploration?
6. Please provide a graphical representation of the proposed framework to facilitate comprehension of the protocol.

Is the study design appropriate for the research question?

Yes

Are sufficient details of the methods provided to allow replication by others?

Partly

Is the rationale for, and objectives of, the study clearly described?

Yes

Are the datasets clearly presented in a useable and accessible format?

Partly

Reviewer Expertise:

My research area is in Bioengineering, focusing on movement analysis for ambient assisted living monitoring and rehabilitation purposes.

I confirm that I have read this submission and believe that I have an appropriate level of expertise to confirm that it is of an acceptable scientific standard, however I have significant reservations, as outlined above.

# Reviewer report: Reviewer response for version 1

**DOI:** 10.21956/openreseurope.18060.r36766

**Published:** 2024-02-21

**Authors:** Ashish D Patel

**Affiliations:** 1 Pandit Deendayal Energy University, Gandhinagar, Gujarat, India

Accepting camera-based active and assisted living technologies involves various factors influencing how individuals, caregivers, and society perceive and adopt these systems. For a good review article, the following points should be included in further study.

 1. Provide a graphical representation of the proposed framework.

 2. Add more review questions.

 3. Discuss how the datasets will be utilized in the study.

 4. Compare the solution with results from the publicly available similar datasets.

 5. Discuss how the labels of the dataset will be created. (manual / automated) 

 6. Discuss the availability/scalability of the provision for intrusive / non-intrusive hardware.

 7. Is there a provision for data fusion? (conventional sensor data and vision sensor data)

 8. What about computational complexity? (any provision for federated learning)

Is the study design appropriate for the research question?

Partly

Are sufficient details of the methods provided to allow replication by others?

Yes

Is the rationale for, and objectives of, the study clearly described?

Yes

Are the datasets clearly presented in a useable and accessible format?

Partly

Reviewer Expertise:

Ambient Intelligence, Assisted Living Facilities, Wireless Sensor Networks

I confirm that I have read this submission and believe that I have an appropriate level of expertise to confirm that it is of an acceptable scientific standard.

# Reviewer report: Reviewer response for version 1

**DOI:** 10.21956/openreseurope.18060.r36424

**Published:** 2024-01-03

**Authors:** Wei Qi Koh

**Affiliations:** 1 The University of Queensland, Saint Lucia, Queensland, Australia

Thank you for the opportunity to review this paper titled “Barriers and facilitators to older adults’ acceptance of camera-based active and assisted living technologies: a scoping review protocol”. Overall, it was a great pleasure to review this paper. This is a very well written protocol, and it is evident that the review process was very meticulously thought through. The authors also demonstrated excellent research transparency. I have a few comments for the authors’ consideration:

- The authors well justified the rationale for this study, stating that they did not find similar reviews on the Cochrane, JBI and MEDLINE databases. While it is commendable that the authors ensured that this study is not a repetition of previous/similar studies, it may be worth considering doing a literature review of other reviews synthesising barriers and facilitators to the acceptance of AAL or other digital technology.

 

- Using the TDF as a conceptual framework is reasonable – is there a reason why the authors chose the TDF instead of specific technological acceptance models (e.g., TAM, UTAUT)?

 

- A qualitative evidence synthesis could have been undertaken in place of a scoping review, given the aims of this study. Could the authors clarify why they did not choose a QES approach?

 

- Narrative synthesis – will the authors use any existing guidance to guide the narrative synthesis of the findings? E.g., Popaye et al. If so, it would be great to provide a brief explanation of the process moving forward.

Is the study design appropriate for the research question?

Yes

Are sufficient details of the methods provided to allow replication by others?

Yes

Is the rationale for, and objectives of, the study clearly described?

Yes

Are the datasets clearly presented in a useable and accessible format?

Yes

Reviewer Expertise:

Evidence synthesis, digital technology, aged care, implementation science

I confirm that I have read this submission and believe that I have an appropriate level of expertise to confirm that it is of an acceptable scientific standard.

## References

[1] : Guidance on the conduct of narrative synthesis in systematic reviews. A product from the ESRC methods programme dust. *https://www.lancaster.ac.uk/media/lancaster-university/content-assets/documents/fhm/dhr/chir/NSsynthesisguidanceVersion1-April2006.pdf* .2006;
